# Heard, not judged: Adolescents’ perspectives on quality of healthcare following self-harm

**DOI:** 10.12669/pjms.42.9.20335

**Published:** 2026-09

**Authors:** Maryam Ayub, Nazish Imran, Amna Rashid, Taimia Ayub

**Affiliations:** 1 Maryam Ayub, FCPS, MRCPsych^,^ Department of Child and Family Psychiatry, King Edward Medical University, Lahore, Punjab, Pakistan; 2 Nazish Imran, FRCPsych, PhD., Department of Child and Family Psychiatry, King Edward Medical University, Lahore, Punjab, Pakistan; 3 Amna Rashid, MBBS. Academic Department of Psychiatry and Behavioral Sciences , King Edward Medical University, Lahore, Punjab, Pakistan; 4 Taimia Ayub, MBBS. Institute of Public Health, Lahore, Pakistan

**Keywords:** Adolescent, Education, Health Personnel, Mental Health Services, Patient-Centered Care, Self-Harm, Self-Injurious Behavior

## Abstract

**Objectives:**

The study aimed to identify the healthcare attributes most valued by adolescents with lived experience of self-harm, and to explore their qualitative experiences and recommendations to inform professional training.

**Methods:**

It was a convergent mixed-methods study done at Child and Family Psychiatry Department, Mayo Hospital, Lahore; over six months from 1^st^ January 2025 to 30^th^ June 2025. Ten adolescents with previous history of self-harm, were recruited via convenient sampling. In the quantitative component, participants ranked five care attributes by personal importance, which was analyzed via mean rank and Borda count. Four participants additionally participated in detailed qualitative interviews which were analyzed using framework analysis.

**Results:**

The mean age of participants was 15.3 years (Range: 13-17). In quantitative sample (n=10), *feeling heard and understood* ranked highest (M = 2.60; Borda = 24), followed by *empathy and non-judgmental attitude*, *clear communication*, and *privacy and confidentiality*. *Involvement in decision-making* ranked last. Through qualitative interviews (n=4), the findings reinforced and deepened these priorities across five themes: therapeutic relationship and emotional safety; communication style and interaction skills; understanding the young person’s experience; support systems, agency, and recovery; and therapeutic techniques and practical interventions. Participants highlighted the harm caused by mocking or dismissive clinician behavior, and the value of calm, personalized, and play-inclusive care.

**Conclusions:**

The adolescents consistently prioritize relational and affective dimensions of care over procedural ones. These findings provide a coherent, patient-centered evidence base for designing healthcare professional training focused on adolescent self-harm.

## INTRODUCTION

Self-harm among children and adolescents is a major global public health concern due to significant psychological distress, morbidity and mortality. Self-harm can be defined as any intentional act that causes injury or poisoning to oneself, irrespective of the presence or absence of suicidal intent.^1^ Self harm is one of the most frequent reasons for emergency hospital admission especially in children and young people.^2^ It has been shown to be one of the most notable predictors of later suicide deaths.^1^ There is a high vulnerability in children and young people of age group of 10-24 years old with suicide being the third leading cause of death in 15-19-year-olds and second leading cause of death in young people aged 15-29 years old.^3^

Deliberate self-harm often begins in adolescence and is commonly associated with emotional dysregulation and maladaptive coping.^4^ Individuals, who engage in self-harm behavior have frequently reported accompanying feelings of worthlessness, guilt, and emotional distress.^4^ Established risk factors include family history of suicide, experiences of bullying, academic stress, lack of friends, parental conflict, exposure to trauma such as sexual assault, and substance use. Thus, healthcare professionals dealing with self-harm in adolescents and children must be comfortable identifying these risk factors and screening for associated mental health conditions such as mood and anxiety disorders.^5^ Unfortunately the experience of presenting to healthcare workers after self-harm may be traumatizing for many patients.^6^ They may experience stigma through derogatory behaviours including name calling like “the attention-seeking type, the dramatic type, the crazy one” and “psych case”.^6^ Such stigmatizing experiences may lead individual to believe they “need to calculate carefully what they say and what they don’t say” to avoid disclosure of previous psychiatric illness or self-harm despite the potential medical risks of omission of important information.^6^ These experiences can ultimately be a large contributory factor in poor treatment adherence and outcomes.^6^

In Pakistan deliberate self-harm presentation remains an under-researched topic as Pakistan doesn’t maintain official suicide statistics.^7^ Only one previous qualitative study has been done in Pakistan in adolescents with self-harm which identified interpersonal conflicts and emotional crises as predisposing factors, alongside post-episode regret.^8^ Compounding this gap, no training program for healthcare professionals for dealing with children and young people who present with self-harm currently exists in Pakistan.^9^ In the absence of such training, professionals may lack the skills and confidence to conduct sensitive risk assessments, respond empathetically, or provide developmentally and culturally appropriate care, a situation that has direct implications for patient safety, engagement with services, and the risk of repeat self-harm. Internationally, training interventions grounded in the lived experiences of service users have been shown to improve clinician knowledge, confidence, and attitudes when responding to self-harm.^10^ However, such training cannot simply be transplanted from other contexts, given that help-seeking behaviours, family dynamics, stigma, and healthcare structures around self-harm in Pakistan differ substantially from those in settings where existing training packages have been developed. This underscores the need for a culturally grounded training module developed specifically for the Pakistani context. The current study forms the first phase of a larger project to co-produce such a training module for healthcare professionals on self-harm

The objective of the current study, was to explore the lived experiences of children and young people who presented with self-harm with specific focus on uncovering priority areas for the development of a culturally responsive healthcare training package for healthcare professionals dealing with self-harm.

## METHODOLOGY

This convergent mixed-methods study was conducted at Child & Family Psychiatry Department at Mayo Hospital, a tertiary care affiliated hospital of King Edward Medical University in Lahore, over a period of six months from 1^st^ January 2025 to 30^th^ June 2025.

### Ethical approval:

Ethical approval of the study was taken from Institutional Review Board at King Edward Medical University (463/RC/KEMU; dated July 13, 2024).

The study employed a convergent (parallel) mixed-methods design, in which quantitative and qualitative data were collected concurrently and analyzed independently before being brought together during interpretation to compare and triangulate the findings.^11^

### Inclusion & Exclusion Criteria:

Adolescents aged 12 to 18 years with history of self-harm within the preceding three months were eligible for participation. Adolescents who declined participation or had intellectual disabilities were excluded.

Ten eligible participants were recruited through convenience sampling from clinical setting. Convenience sampling was used because recruitment was undertaken among eligible adolescents presenting to the clinical service during the study period and depended on their willingness, and that of their parents/guardians, to participate in a study concerning the sensitive topic of self-harm. Written informed consent was taken from both parents/guardians and assent from participating adolescents, in line with ethical best practice. Participation was voluntary, and confidentiality and anonymity were maintained throughout. A distress protocol was in place, with psychological support available to any participant who became distressed during or after participation.

### Quantitative data collection and analysis:

After informed consent, demographic information (age, gender, education status) was collected. Based on attributes identified from previous research, participants were asked to rank five aspects of healthcare according to their perceived importance, assigning a unique rank each, from 1 (most important) to 5 (least important) to each attribute.^8^,^12^ The five attributes were:

- Feeling heard and understood;
- Clear communication;
- Privacy and confidentiality;
- Empathy and a non-judgmental attitude;
- Involvement in decision-making. The ranking exercise was presented as a structured questionnaire, with each attribute provided as a separate item for participants to rank.

Two methods were used to summarize the data. First, mean rank was computed for each attribute across all ten participants, with lower values indicating greater preference. Second, a Borda count was calculated by assigning four points to rank-1 response, three points to rank-2, two points to rank-3, one point to rank-4, and zero points to rank-5, and summing across participants (maximum = 40).^13^ The Borda count was used as a complementary measure to mean rank because it incorporates the full ordinal ranking provided by each participant, rather than considering only the attribute receiving the highest preference. It therefore provides an overall measure of the relative ordering of the five attributes and allows comparison of preferences across the full ranking distribution.^13^,^14^

### Qualitative data collection and analysis:

Of the ten participants who completed the quantitative ranking exercise, four additionally assented to participate in the qualitative component. All the participants who assented to the qualitative component were included in the interviews. Semi-structured interviews were conducted in Urdu language, using a topic guide developed from previous research.^2^,^8^,^9^,^10^,^15^ The questions explored participants’ experiences with healthcare professionals, the competencies they valued in clinical staff, and their vision of ideal care following self-harm. The interviews were facilitated by trained researchers. All interviews were audio recorded with participants’ consent.

The interviews were transcribed verbatim in Urdu and then translated into English by a bilingual member of the research team, fluent in both Urdu and English. To assess translation accuracy, a second bilingual researcher independently back-translated a sample of English transcripts into Urdu. Discrepancies between the original and back-translated versions were discussed and resolved by the research team, consistent with recommended practice for cross-language qualitative research.^16^ Identification numbers were assigned to all the participants and transcripts were anonymized prior to analysis.

Data were analyzed using the five stages of a Framework Analysis. Framework Analysis was chosen over other qualitative approaches, such as thematic analysis, because it accommodates both prior themes derived from the topic guide and themes emerging inductively from the data, and provides a systematic, transparent structure well suited to applied health research with a pre-defined analytic focus.^17^ Data were analyzed independently by two researchers trained in qualitative methods. The five stages involved familiarization with the data, identification of a theoretical framework, indexing, charting and mapping of themes, and finally interpretation of themes.^17^ Researchers immersed themselves in the data through repeated reading of transcripts, reviewing audio recordings, and studying notes. A conceptual thematic framework was developed from three sources: issues identified a priori from the topic guide, themes emerging from participants’ accounts, and analytical patterns identified through recurring views and experiences.

Themes were systematically applied to the transcripts through indexing, with categories and subcategories documented. Data were subsequently charted by extracting relevant material from its original context and reorganization into summary tables , allowing patterns across participants to be examined while retaining the context of individual accounts. The final stage involved mapping and interpretation, bringing together the key characteristics of the data to develop an overall understanding of participants’ experiences and perspectives.

Several strategies were used to enhance the trustworthiness of the findings.^18^,^19^ Credibility was supported through researcher triangulation, with two researchers independently developing and comparing the coding framework and resolving discrepancies through discussion and consensus. Transferability was addressed by providing a detailed description of the study context, participant characteristics, and sampling approach, enabling readers to assess the applicability of findings to other settings. Dependability was ensured by maintaining a clear audit trail of analytic decisions and through consistency checks between researchers. Confirmability was supported through reflexive discussion among the research team throughout data collection and analysis, helping to minimize the influence of individual researcher bias on interpretation. Interviewing researchers also maintained reflexive notes throughout data collection and analysis.

Given the small convenience sample and the exploratory focus of this first phase of a larger project, formal thematic saturation was not the primary objective. The analysis instead focused on identifying recurring patterns, areas of convergence and divergence, and the range of experiences represented within the available accounts. As a further measure of researcher triangulation, the coding framework and emerging themes were reviewed by two senior members of the wider research team who were not involved in the original coding, providing an additional opportunity to challenge interpretations and enhance the confirmability of the findings.

### Integration of quantitative and qualitative findings:

Quantitative and qualitative findings were analyzed independently and subsequently integrated during interpretation. The quantitative ranking results were compared with themes emerging from the qualitative interviews to identify areas of convergence, complementarity and divergence. In particular, participants’ ranked priorities were considered alongside their narratives regarding experiences with healthcare professionals and their expectations of care following self-harm. This integration enabled the quantitative ranking findings to be interpreted in the context of participants’ lived experiences and provided a more comprehensive understanding of adolescents’ perspectives on the quality of healthcare following self-harm.

## RESULTS

There were ten participants in quantitative portion of study (Table-I) among whom four participants assented to qualitative interviews.

**Table-I:** Demographic characteristics of participants in quantitative study

Sr	Characteristic	Total (N=10)
1	***Age***	
	Mean (SD)	15.3 (1.2)
	Range	13-17
2	***Gender, n (%)***	
	Male	4 (40%)
	Female	6 (60%)
3	***Education Level***	
	Primary school or below	2 (20%)
	Secondary school	4 (40%)
	High school	4 (40%)

In quantitative results, feeling heard and understood emerged as the most preferred attribute with a mean rank of 2.60 and a Borda score of 24 points; it also received the most first-place votes. Empathy and a non-judgmental attitude ranked second, followed by clear communication and privacy and confidentiality Involvement in decision-making ranked last and was the only attribute to receive no first-place votes, (Table-II).

**Table-II:** Participant-Ranked Importance of Therapeutic Attributes.

Rank	Item	Attribute	M rank	SD	Median rank	Borda Score	Rank 1 (n)
1	A	Feeling heard and understood	2.60	1.51	3.0	24	4
2	D	Empathy and non-judgmental attitude	2.80	1.48	3.0	22	2
3	B	Clear communication	3.00	1.56	3.5	20	3
4	C	Privacy and confidentiality	3.10	1.45	3.0	19	1
5	E	Involvement in decision making	3.50	1.27	4.0	15	0

M rank = mean rank (lower values indicate greater preference); SD = standard deviation; Median rank = median rank (lower values indicate greater preference); Borda Score = sum-based preference score (higher values indicate greater preference); Rank 1 (n) = number of participants assigning highest priority.

In qualitative data , four participants consented and then framework analysis was used for themes pertaining to training module for healthcare workers. Participant P 1(a 16 years old male, with one suicide attempt and multiple episodes of deliberate self-harm) and participant P 2 (also 16 years old male, with deliberate self-harm including one suicide attempt) were both studying in secondary schools. Participant, P 3, was a 15 years old female with multiple episodes of deliberate self-harm, and currently enrolled in high school. Participant , P 4, was 15 years old female with multiple episodes of deliberate self-harm with education till primary school, (Table-III, Table-IV).

**Table-III:** Themes and Subthemes Derived from Framework Analysis of Qualitative Interviews with Children and Young People Presenting with Deliberate Self-Harm.

Theme	Sub-Theme	Sample Quote	Participant ID
Therapeutic Relationship & Emotional Safety	Being Heard & Understood	“She [psychologist] was hearing what I was saying … She was understanding what I was saying and advising me accordingly.”	P 2
	Listening deeply	“When you are listening to patients, I think you do listen — but you should listen even more so patient can get out what they are thinking.”	P 3
	Feeling judged or dismissed	“One senior doctor made fun of my suicide attempt which kind of hurt me… He used the phrase ‘phir koi masla aya to phir latak jao gay’ (next time you get stressed you’ll hang again) multiple times. He just counselled as if he was arguing, and it made me angry.”	P 2
	Personalized communication	“They should talk to patient by their name. The most important thing for a person is their name. When you address them by their name, they will feel better.”	P 3
	Confidentiality & Safe Space	“A third party like doctors should tell them politely ‘we are here for you, you can tell us openly what is the problem’.”	P 3
	Trusted therapeutic Attachment	“First 2 days, I didn’t like her at all. But slowly she handled me/ built relationship.”	P 2
Communication Style & Interaction Skills	Calm, Non- Confrontational Tone	“They should talk with softly… It’s not like we have come to dump our anger on them.”	P 1
	Polite Conduct as a Baseline Expectation	“Everyone is polite here… Nurses are all there, politely. Everyone should talk politely and should not talk angrily.”	P 1
	Giving choices and flexibility	“Instead of saying ‘if you don’t do this you won’t get better’ — say ‘if this is happening to you, maybe you should try this and it will improve’.”	P 2
		Yes (giving choices is better) because she (psychologist) had agreed I can do it (the task) tomorrow. So it felt good and I ended by doing it the same day.”	P 4
	Gentle Firmness	“They should be a bit strict — but lovingly. Patient shouldn’t feel they are being scolded but they should make them do the work.”	P 4
	Staying Focused — One Topic at a Time	“When Ms. .. (psychologist) used to interrupt to remain on one topic, it was good so I can solve one topic (problem).She counselled me to stay on one topic and that is a good thing. This is something that you guys should do.”	P 3
	Sensitivity to Self-Harm Presentation	“When someone who has done self-harm presents, they don’t want to be scolded.”	P 3
Understanding the Young Person’s Experience	Knowing the Patient’s History	“They should know back history of the child, what phase the child has passed through, what’s the reason he is doing self-harm. That’s how they can help him get better.”	P 2
	Helping Patients Understand Their Own Treatment	“I am a patient so sometimes I don’t understand why I am doing this. So they (doctors) should help them figure out. That is very helpful.”	P 3
	Talking at the Child’s Level	“Doctors should stay to understand why he is depressed… they should talk at the level of child. If doctors do truly try to understand, the child will be able to explain better.”	P 2
Support Systems, Agency & Recovery	Balanced Support	“They need a helping hand. But also not so much that it seems artificial. They need someone to be their helping hand on a normal basis — not too little and not too much.”	P 3
	Emotional Acknowledgement	“If the doctor just says, ‘I understand’, ‘I understand your pain’ — this alone can be a big thing for patient.”	P 3
	Patient’s Role in Recovery	“In mental health no one can make them better. No medicine can (by itself) . They only have 50% role — the rest 50% is patient themselves .If you support patient — that your fight is with yourself — you can remind them they’ll try to win.”	P 3
	Role of family	“Parents should be available if they can… or any other available close family members .”	P 2
	Clinician Resilience	“Patient can feel bad over everything. That is not doctor’s fault. Patient can get agitated (irritable) over anything — they can deteriorate over anything. Doctors shouldn’t be disheartened. I know doctors put a lot of effort on one patient and they may be ill again… if they give up, what will happen to the patients.”	P 3
	Peer support	“ I share everything with her (my cousin) and that helps.”	P 2
Therapeutic Techniques & Practical Interventions	Breathing and Distraction Exercises	“The therapist helped me a lot in doing… she told me how to divert attention (exercises), she told me breathing exercises.”	P 2
	Reliable clinician availability	All doctors, nurses, are there for you. They are present. . They all want (you to) get better.”	P 2
	Play as part of Therapy	‘If the person is too depressed, maybe play with them first. “ (Nurses) were good. They played badminton with me. Those who play (with me), I find it easier to bond with”	P 1

**Table-IV:** Integration of Quantitative Priorities and Qualitative Themes

Quantitative Attribute	Corresponding Qualitative Theme(s) (Subtheme(s)
A. Feeling heard and understood (Rank 1)	Therapeutic Relationship & Emotional Safety (Being Heard & Understood; Listening Deeply)
D. Empathy and non-judgmental attitude (Rank 2)	Therapeutic Relationship & Emotional Safety (Feeling Judged or Dismissed) (inverse);
	Communication Style (Calm,Non-Confrontational Tone; Sensitivity to Self-Harm Presentation)
B. Clear communication (Rank 3)	Communication Style & Interaction Skills (Giving Choices and Flexibility, Staying Focused)
	Understanding the Young Person’s Experience (Talking at the Child’s Level
C. Privacy and confidentiality (Rank 4)	Therapeutic Relationship & Emotional Safety (Confidentiality & Safe Space; Trusted Therapeutic Attachment)
E. Involvement in decision-making (Rank 5)	Support Systems, Agency & Recovery (Patient’s Role in Recovery; Balanced Support)
	Communication Style (Giving Choices and Flexibility)

## DISCUSSION

Our study is first of its kind from Pakistan to focus on exploring what young people value in care by the healthcare staff treating them after an episode of self-harm. The qualitative and quantitative aspect provided valuable information for co-production of training module for healthcare professionals. The young people were eloquent in telling their exact needs while being empathetic to difficulties of their families and healthcare staff in understanding the complexities self-harm. The importance of therapeutic relationship and emotional safety in both qualitative and quantitative data aligns with previous literature that non-judgmental, validating relationship and interpersonal skills are central to how young people experience care and ultimately significantly affect outcomes and self-harming (Table-IV).^12^,^20^ Lowest ranking of involvement in decision making may signify cultural patrilineal trends of autonomy in Pakistani family-oriented society. The physicians are regarded as authoritarian figures and medical paternalism is often accepted and even valued by patients and their families.^21^ It’s also essential to avoid dismissive, mocking and confrontational styles as it alienates young people very quickly and may worsen prognosis.^6^ The training module should accordingly be structured to foreground rapport-building and active listening as foundational elements.

Personalized communication and explicit confidentiality assurance signal respect and create psychological safety. Generic scripted talk, procedural reassurance and being “ *just another number that just needs to get shipped out of here”* can feel dismissive, while personalization and staff being their authentic selves encourage a genuine connection, whereby increasingly intimate and sensitive discussions can occur.^22^ Structure and firmness are important. But equally important is understanding power of curiosity and flexibility. De-escalation and rapport building are essential before delving into detailed self-harm inquiries. Structured rapport-building strategies, such as showing genuine interest and engaging in informal conversation, are important and have previously been shown to improve clinical encounters and patient cooperation, even when patients are initially guarded or resistant.^23^ Accordingly, the module should incorporate explicit personalized communication skills training alongside structured strategies for rapport-building.

Understanding the young person’s experience is ultimately an ongoing process. The healthcare professional must gather contextual and corroborative history as relying solely on the young person’s or the family’s account can each introduce gaps.^24^ Encouraging age-appropriate self-exploration and disclosure is consistent with evidence-based interventions for adolescent self-harm.^25^ When it comes to therapeutic techniques , specific doable skills like breathing exercises and attention training are valued by young patients. Guidance for healthcare professionals should also highlight the value of play-based engagement across developmental stages. Young people recognize that recovery is a shared endeavor hence role of personal agency can be used to motivate patients. Parents and family engagement are essential, not only in supporting young people’s recovery but also helping families manage their own distress and emotional impact.^22^ Equally important is addressing professional resilience and compassion fatigue to avoid being discouraged by natural fluctuations in patient state. Collectively, these findings indicate that the training module should equip healthcare professionals with a flexible, developmentally informed toolkit, combining structured rapport-building and active listening with concrete therapeutic techniques, family engagement strategies, and attention to their own emotional resilience, to meet the varied needs of young people following self-harm. The clinical implications of this study, while mainly intended for the development of a training module for healthcare professionals, go beyond that and provide a useful guidance for healthcare professionals in LMICs like Pakistan about what do the children and young people want in the difficult time following self-harm, (Fig.1).

**Fig.1:**
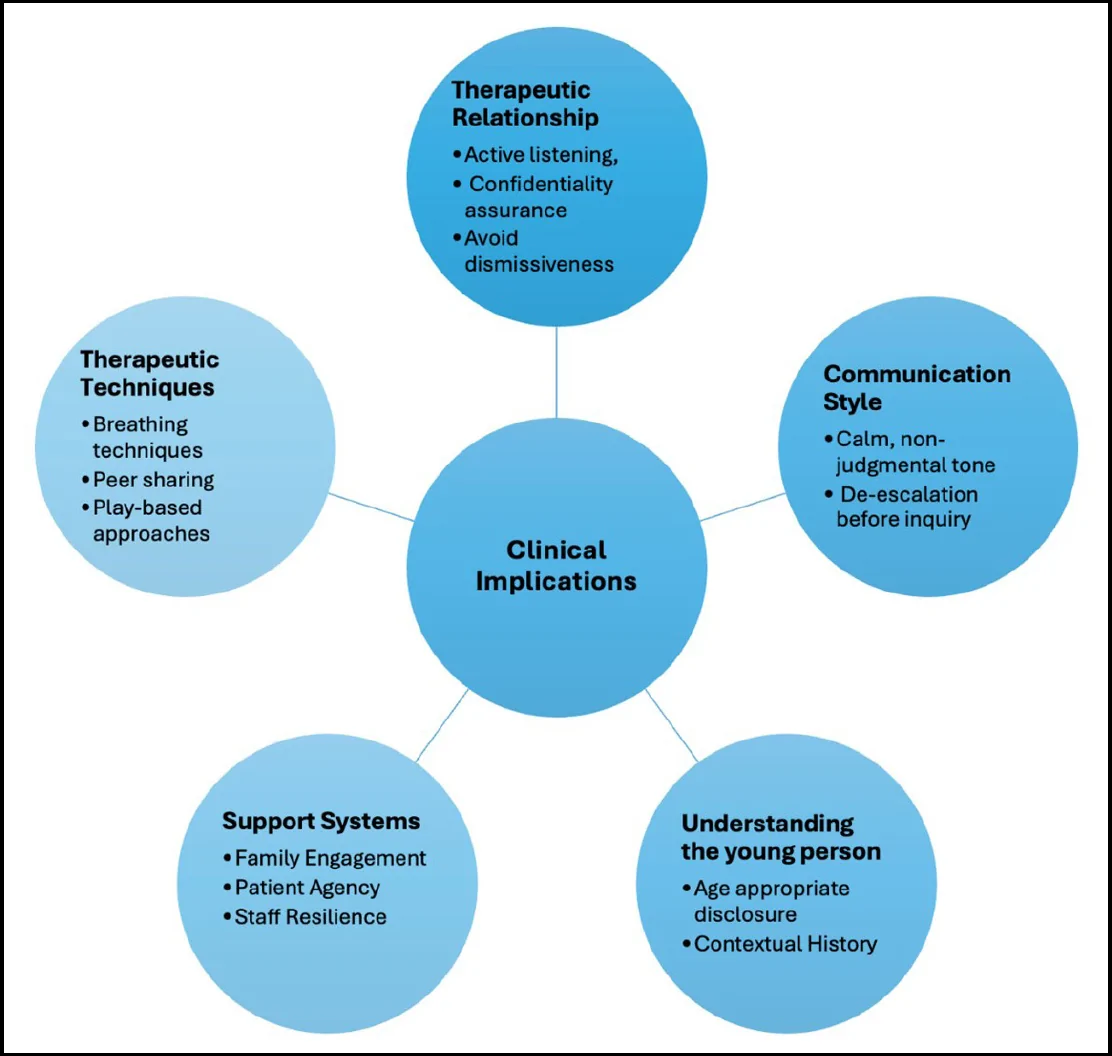
Clinical Implication of Interviews with Children and Young people who presented with self-harm.

### Limitations

The sample size was very small (n=10 quantitative; n=4 qualitative) which may limit statistical power and thematic saturation. Furthermore, convenience sampling from only one urban hospital setting limits demographic diversity through bias and limits generalizability as all participants were already actively engaged with psychiatric services. Social desirability or recall bias may also have influenced responses given the clinical setting. Despite these limitations, the study had significant strengths.

### Strengths of the study:

It’s first study of its kind in Pakistan by centering account of children and young people with lived experience of self-harm in co-production of training module for healthcare workers. The aim was to ensure the training module reflected the experiences and priorities of young people, rather than to achieve statistical or thematic generalizability. Furthermore, the mixed-methods design, combining quantitative ranking with qualitative interviews, allowed triangulation of findings, strengthening credibility. Framework analysis was well-suited to the applied goal of module development, and ethical practice was robust, including dual consent, anonymization, distress protocols, and independent analytical review.

## CONCLUSION

It is reiterated that children and young people consistently prioritize relational and affective dimensions of care over procedural ones. These findings provide a coherent, patient-centered evidence base and will directly inform the next phase of the wider project: co-production and pilot-testing of the healthcare professional training module focused on adolescent self-harm as intended. Future work should further extend recruitment across multiple sites and a broader age range, include adolescents and children who have not engaged with psychiatric services, and formally evaluate the module’s impact on clinician knowledge, confidence, and attitudes, alongside implementation barriers in real-world LMIC clinical settings.

### Authors’ Contributions:

**MA and NI:** Participated in conceiving the design of the study and coordination of project.

**MA, NI, AR and TA:** Participated in literature review, data collection, data analysis and preparing the manuscript.

**MA and NI:** Helped in critical revision and finalizing the manuscript.

**MA**: Responsible and accountable for the accuracy and integrity of the work.

All authors have read , revised and approved the final manuscript.
